# Preschool- and childcare center-based interventions to increase fruit and vegetable intake in preschool children in the United States: a systematic review of effectiveness and behavior change techniques

**DOI:** 10.1186/s12966-023-01472-8

**Published:** 2023-06-03

**Authors:** Faten Hasan, Andy V. Nguyen, Amaya R. Reynolds, Wen You, Jamie Zoellner, Amanda J. Nguyen, Damon Swift, Sibylle Kranz

**Affiliations:** 1 Department of Kinesiology, University of Virginia, Charlottesville, VA 22903 USA; 2 Department of Human Services, University of Virginia, Charlottesville, VA 22903 USA; 3 Department of Public Health Sciences, University of Virginia, Charlottesville, VA 22903 USA

**Keywords:** Nutrition Education, Behavior Change Theory, Diet quality, Eating behavior, Behavioral intervention, Nutrition intervention, Repeated exposure

## Abstract

**Background:**

Fruit and vegetable (FV) consumption in children in the United States (US) is very low. Adequate FV consumption is required for proper development during childhood, and dietary habits are established during preschool-age and tend to persist into adulthood. As most U.S. preschool-aged children attend childcare or preschool, this may be an opportune time and setting to conduct interventions to improve FV intake. These interventions should be based in theory and use behavior change techniques (BCTs) to explain mechanisms for expected change. To date, no published reviews have examined the effectiveness of childcare- or preschool-based FV interventions in preschoolers and their use of theoretical frameworks and BCTs.

**Methods:**

This systematic review was completed adhering to the Preferred Reporting Items for Systematic Reviews and Meta-Analyses guidelines. Inclusion criteria were randomized controlled trials (RCTs) published between 2012 and 2022 of interventions to improve diet or FV intake in preschoolers (aged 2–5 years) in childcare or preschool-settings. A search of four databases was conducted between in September 2022 using search terms pertaining to the study’s primary aim (FV consumption), age group (preschool-aged), settings (US childcare or preschool settings), and study design (RCT). Additional criteria were objective measures of FV consumption or skin carotenoids, as a proxy for FV intake. Included studies were narratively synthesized based on intervention type, measured effect, and use of theory and BCTs.

**Results:**

The search resulted in six studies that reported on nine interventions. Overall, six interventions increased FV intake, of which five used nutrition education and one manipulated the feeding environment. Among the three interventions with no measured effect, two manipulated the feeding environment and one used peer modeling. Effective studies used at least three BCTs, though no pattern was observed between use of theory or BCTs and intervention effect**.**

**Conclusions:**

While several studies have shown promising results, the limited number of studies identified in this review highlights key gaps in this field: there is a need for studies to test FV interventions in US childcare settings that use objective measures of FV intake, directly compare intervention components and BCTs, are theory-based, and assess long-term behavior change.

**Supplementary Information:**

The online version contains supplementary material available at 10.1186/s12966-023-01472-8.

## Introduction

Promoting fruit and vegetable (FV) intake in children is critical to support proper brain and body development [1–3] and to establish healthy dietary habits that persist over the life course [4, 5]. Despite the long-standing explicit guidelines and evidence regarding the importance of FV intake, average FV consumption amongst all US children (2–18 years old) remains low: only 40% and 7% meet the recommended intake for fruits and vegetables, respectively [6]. The current average intake for children is only 0.9 cup equivalents of each (60% of recommended), with consumption levels that decline with age and are restricted in variety relative to recommended guidelines [7]. Thus, in support of public health, it is important to develop strategies to improve FV intake in children.

Preschool age children begin developing their own dietary habits by gaining autonomy over their food choice [4, 5], evident in the decline in FV intake as children transition from preschool age to school age [8–10]. Thus, preschool age (2–5 years old) may be an optimal time for a dietary intervention to promote FV intake throughout the lifespan. However, there are key gaps and inconsistencies present in diet research that will be discussed below.

Consideration of the measurement tool in diet research is particularly important in young children because they are unable to accurately report their own intake [11, 12]. Parent-reported measures, while most frequently used in research [13–15], have been repeatedly shown to be subjective and prone to recall and reporting bias [16, 17]; this is especially problematic when parents are asked to report on periods of time for which they are not directly responsible for child feeding, such as during childcare hours [18, 19]. As such, use of objective observation measures is critical with this age group [20, 21]. An additional objective measure specific to FV intake is the skin carotenoid level [22–26]. When FVs are consumed, the carotenoids in the FVs are absorbed and then deposited in various tissues including the skin [27], which can be quantified using reflection spectroscopy [17] to objectively measure FV intake within the previous two to four weeks [26, 28, 29]. Due to the discrepancies associated with the different techniques used to measure dietary intake, reviews should distinguish between studies with subjective and objective measures to adequately evaluate the validity of a large proportion of this body of work.

The setting of dietary interventions in children is another significant consideration. In 2019, nearly two-thirds (64%) of 3–5 year old children in the US were enrolled in childcare or preschool with 64.7% of all enrollments being full-time [30]. Thus, childcare services have a large influence on children’s development in the US [31], and this may be an efficient avenue to effectively influence children’s behavior, namely dietary behavior [32–34]. In evaluating intervention effectiveness, it is therefore vital to consider the setting of the interventions to avoid extrapolating evidence for intervention effectiveness to other contexts.

Behavior change techniques (BCTs) are the intervention components regarded as the “active ingredients” within behavior change interventions. It is often helpful to examine the use of BCTs within interventions aimed at changing dietary behaviors to assess the mechanism by which interventions may be effective at causing the behavior change [35]. Given the heterogeneity in intervention techniques used in dietary interventions, evaluation of BCTs in reviews and meta-analyses may provide important insight on the underlying intervention components that may be at play [35–37].

Use of theory in developing an intervention is another metric that can be examined within interventions that target behavior change. This metric evaluates how exactly a specific theory and its concepts are utilized to tailor intervention techniques and components and allows for a better understanding of why an intervention is effective or ineffective. This knowledge may then be evaluated in systematic reviews and applied to the refinement of an interventions to better target the tenets of the theory [38, 39].

A recent systematic literature review by Hodder et al. [40] of FV intake in children five years and younger identified 80 trials reporting a large variety of interventions to promote FV intake in preschool-aged children globally. This review included interventions conducted in all settings and using various intervention methods, though it did not distinguish between subjective and objective measurements of food intake or evaluate BCTs and use of theory in the included studies. The main implications drawn from these findings is that more pragmatic studies are needed to test the interventions, and that these interventions should be based on BCTs and theoretical frameworks that may explain the mechanism by which these interventions may change children’s dietary behaviors related to FV intake [41].

In 2012, Hendrie et al. [42] published a systematic review to explore the use of BCTs in home- and school-based interventions for the prevention of childhood obesity involving children and parents. More specifically, they compared the number and type of BCTs used in effective and ineffective studies and reported that effective studies incorporated more BCTs than ineffective studies (median of 10 versus 6.5). However, this review was not specific to interventions aimed at improving FV intake, so we are unable to determine whether these findings are applicable to FV interventions specifically.

In 2017, Hendrie et al. [43] used a similar technique to evaluate interventions to improve vegetable intake in children and found that the BCTs “Repeated exposure”, “Provision of staff training,” and “Planning for social support or change,” were associated with effective behavior change. This review only examined interventions conducted in home or community settings and, therefore, these results may not be extrapolated to interventions conducted in other settings, such as childcare centers [53]. Similar to the review by Hodder et al., these two reviews are limited by their inclusion of subjective measures of dietary change, and lack of evaluation of use of theory within the included studies.

To our knowledge, there are currently no published reviews that evaluate both the effectiveness of FV interventions specifically in childcare- or preschool-based settings in the US, and the use of theoretical frameworks and BCTs within these studies. This is a critical gap as comparing different types of interventions, exploring whether use of theory and BCTs moderate effectiveness, and summarizing the level of evidence is critically needed to develop effective interventions in the future. Therefore, the purpose of this review was to systematically identify published randomized controlled trials (RCTs) evaluating childcare- or preschool-based interventions designed to increase objectively measured intake of fruits, vegetables, or both, in preschool children (aged 2–5 years) in the US and to summarize their methods and results. This review also aimed to identify the use of theoretical models and BCTs in each study and to assess their effectiveness in improving FV intake.

## Methods

### Search strategy

This systematic review utilized the Preferred Reporting Items for Systematic Reviews and Meta-Analyses (PRISMA) guidelines and was pre-registered with PROSPERO International Prospective Register of Systematic Reviews (ID: CRD42022350953). Studies were identified using CINAHL, PubMed, Web of Science, and MEDLINE (Ovid) between August 15 and September 4, 2022. The following search terms, using Boolean operators and MeSH terms (PubMed), were used: (fruit OR vegetable) AND (intake OR consumption) AND (“young children” OR preschool OR pre-kindergarten) AND (preschool OR center* OR childcare* OR daycare*) AND (intervention* OR program OR “nutrition education”). Additional search hedges included in the search were designed to include only randomized-controlled trials and studies conducted within the US. The search was restricted to articles published in English between January 2012 and September 2022. The reference list of included articles were reviewed to identify additional relevant articles. Search strategies were reviewed by a university librarian.

### Grey literature

The risk of publication bias was evaluated by examining preprints and unpublished studies from clinical trial registries and dissertation/theses, using a google search tool to limit results to “.gov,” “.org”, and “.edu” sources, ClinicalTrials.gov, ProQuest Dissertations and Theses Global database (unpublished theses), and MedRxiv (pre-prints of relevant studies). The search terms used for the grey literature search were “fruit and vegetable interventions in preschoolers in the United States.” The search using the ProQuest Dissertations and Theses database was limited to scholarly journals and dissertations and theses, only English and in the US, and within the last 10 years. The search using the MedRxiv database used the previously stated Boolean search terms and was also limited to the last 10 years.

The risk of outcome reporting bias was assessed by comparing planned outcomes in the Methods section with reported outcomes to identify any missing outcomes. Two researchers (FH and AVN) independently screened the titles and abstracts of all articles based on the inclusion and exclusion criteria stated below. Any discrepancies between reviewers were resolved by discussion until consensus was reached. If necessary, a third reviewer (SK) was consulted.

### Eligibility criteria

Eligible studies were randomized controlled trials (RCTs) conducted in the US that utilized an intervention to improve fruit and/or vegetable intake in preschoolers (children 2–5 years old), were conducted in preschool or childcare settings, and examined the magnitude of change from baseline (between an intervention and control group) of the number of servings, portions, or grams of FVs consumed or changes in skin carotenoid levels used as a proxy for FV intake. We restricted our inclusion criteria study reports to interventions in the US to allow for comparison between studies that are based on the uniform national recommendations/guidelines, similar food supply, similar access to federal nutrition programs (WIC and SNAP) and known cultural differences in dietary patterns. For instance, the United Kingdom National Health Services (NHS) dietary guidelines for two- to five-year old children are: “should gradually move to eating the same foods as the rest of the family in the proportions shown in the Eatwell Guide [44],” with no specific consumption amount or type of FV specified during childhood. The Eatwell Guide specification for which FV to consume is different from the US guidance, for example, potatoes are not counted as vegetables. Only studies published within the previous 10 years were considered for inclusion to ensure recency of data and to reflect changes in early childhood education approaches. For example, the Mere Exposure hypothesis, first developed in 1968 and later applied to the context of children’s dietary behavior, was considered the cornerstone method of improving children’s diet until relatively recent studies found that it may not be applicable to all children [45] and results may not be sustainable [46] and researchers modified their approach. Only studies in which FV intake was measured objectively were included, and FV intake or diet must have been measured as one of the primary aims. Studies that reported only subjective measures of FV intake were excluded due to the lower validity of subjective dietary measures. Hence, those that used self-reported measures, such as dietary recalls or caregiver questionnaires, were excluded. Interventions that aimed to improve overall diet quality with fruit and/or vegetables as a component were also included; interventions whose primary aim were not diet related, such as those aiming to produce weight loss, were excluded due to the potential for confounding.

The population being studied included preschool children, parents, guardians, caregivers, and professionals responsible for the care of preschool children. Studies were included if they combined both child- and parent-targeted interventions. Studies were excluded if the interventions *only* targeted parents or caregivers of preschoolers, such as only parent-focused nutrition education interventions. Studies focused on populations with special developmental considerations, such as autism spectrum disorder, were also excluded because these children have been shown to respond differently to dietary interventions [47–49].

### Data extraction and synthesis

Data selection and extraction were conducted with the use of Covidence, a program developed for conducting systematic reviews and meta-analyses. Studies were independently reviewed by two reviewers (FH and AVN) and selected for inclusion using a pre-specified form with the inclusion and exclusion criteria explicitly stated and reviewed by a professional expert in childhood nutrition (SK). Disagreements about article classifications between reviewers were resolved by discussion and consensus between the two reviewers (FH and AVN). A third reviewer (SK) was consulted, if necessary. A data extraction form was developed under supervision of an expert in the topic (WY) and piloted on five randomly selected studies that met inclusion criteria. Data was then extracted in duplicate by the first author (FH) and two additional authors (AVN and ARR) to ensure duplicity.

Outcome data were extracted using mean difference in fruit and/or vegetable intake, where outcomes were reported in grams, and standardized mean difference, where outcomes were reported using a different method (grams per kilogram of body weight, grams per total energy intake, servings). The results of statistical analyses were extracted including, but not limited to, t-tests, analysis of variance, analysis of covariance, and linear and mixed-model regressions. For studies that reported multiple timepoints for fruit and/or vegetable intake, the data for each timepoint was extracted separately. For studies that did not report effect size, effect size estimates (Cohen’s *d*) were calculated by the first author using either means/standard deviations of each group or reported t-test values. We were unable to estimate effect size for two interventions due to lack of reported standard deviations [50]. We also coded for additional study characteristics, if applicable to the study, including: 1) information pertaining to the study design, setting, length, frequency, description and length of interventions, outcomes measured and measurement tools used to obtain these measurements, 2) sample size, mean age of participants or proportion of children within provided age groups, sex of participants, 3) identification and classification of behavioral or cognitive theories and/or models used, 4) identification and classification of BCTs used, 5) predictors and/or confounders of response to the FV intervention, and 6) cost-effectiveness of the intervention, if applicable. The additional variables were evaluated on a case-by-case basis and compared to the usual care and/or comparator(s) defined within the intervention.

The use of BCTs were identified within studies and coded accordingly using a standardized taxonomy of behavior change techniques [36]. This taxonomy consists of 93 unique BCTs grouped into 16 domains. BCTs used within studies were coded in duplicate by the first author (FH) and two independent reviewers (AVN and ARR). Interclass correlation coefficient was used to establish intercoder reliability of BCTs present and absent, as well as number of BCTs used in each study. Any discrepancies were then resolved by consensus amongst all three coders.

The use of theory within studies was also evaluated by the first author (FH) using a Theory Coding Scheme developed by Michie and Prestwich in 2010 [38]. This taxonomy consists of 19 items; items 1–6 evaluate if theory is mentioned, whether it was used to select participants, and whether it was tailored to participants, items 7–11 evaluate whether the relevant theoretical constructs were explicitly targeted and all intervention techniques are linked to a specific construct, and items 12–19 assess whether the theory was adequately measured within the intervention and explains the changes observed, and whether the theory was refined based on the intervention’s outcomes. This review aimed to identify the use of theory in the development of interventions, rather than how well the specific theories were able to yield results. Hence, items 12–19 were not evaluated in this review [39]. Items 1–11 were scored based on whether they were present within the paper and were summed to yield a Use of Theory score ranging from 0 to 11, with higher scores indicating greater use of theory [39]. For studies for which a separate publication was available to describe the intervention development or methodology, we used that publication to assess the intervention’s Use of Theory score.

The included studies were organized into subgroups by type of intervention (e.g. repeated exposure, nutrition education). Although intervention types were not directly compared to one another due to heterogeneity between studies, each subgroup was evaluated as a group based on the level of evidence, patterns in the measured effect (mean difference in fruit and/or vegetable intake), and any adverse effects unique to that intervention type. Information regarding the maintenance or sustainability of the behavior change was evaluated by assessing data of a post-intervention follow-up period, if applicable, within each included study. Following narrative synthesis of the results, the additional outcomes were evaluated in the context of the intervention and its overall desired effect.

### Quality assessment

Risk of bias within studies was examined using the Cochrane risk of bias assessment tool [51]. This tool evaluates randomization, deviations from the intended interventions, outcome data, measurement of outcome, and selection of the reported results. Two researchers (FH and AVN) individually assessed the included studies for risk of bias using this tool. The risk of bias was judged within each domain and overall risk-of-bias as 'low risk', 'some concerns', 'high risk', or 'unclear risk'. Any discrepancies between reviewers were resolved by consensus between the two reviewers.

## Results

### Search results

**Fig. 1:**
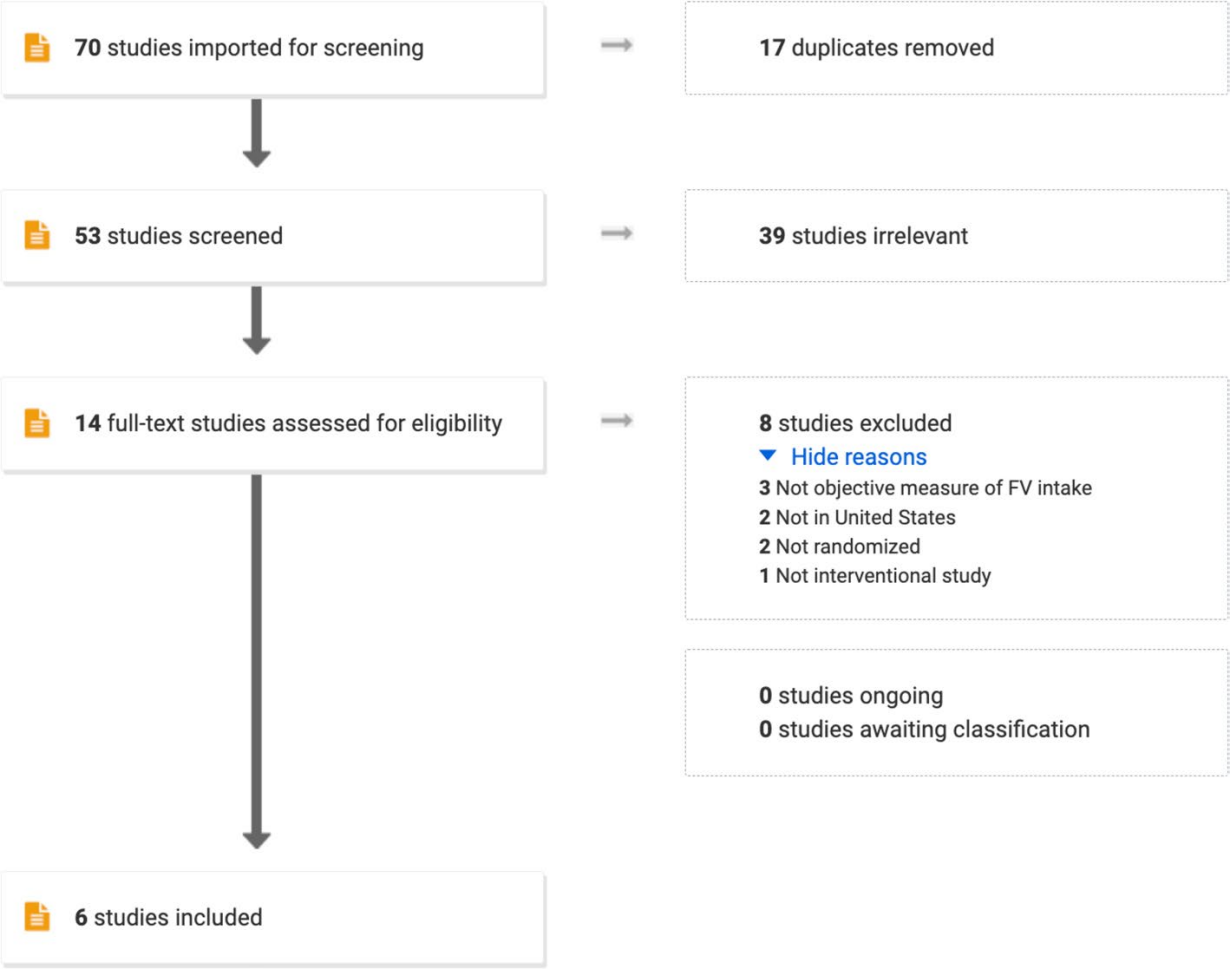
PRISMA flow diagram of literature search

A total of 70 studies was identified during the initial search. After removing duplicates, 53 remained and were screened by title and abstract using the inclusion and exclusion criteria. No additional studies were identified using backward and forward snowballing. No studies were identified in the grey literature. After title and abstract screening, 14 studies were screened as full text, and eight were excluded for various reasons, such as not measuring FV intake objectively (ie. using self-reported measures of dietary intake), not being conducted in the US, and not RCTs. The final review included six papers that reported on nine unique interventions from 2012 to 2022 (Fig. 1). Studies that reported on multiple intervention groups were analyzed as separate interventions.

### Study characteristics

Study designs included cluster- (six interventions), crossover- (two interventions) and individual- (one intervention) RCTs. Interventions ranged from a single day to 12 weeks, with a frequency of two to five days per week. The mean total intervention days across all interventions was 20.33 days, with a range of 1 to 40 days. Only two studies (two interventions) included a follow-up period, which included seven days [52] and three months [53] post-intervention. The remaining four studies (seven interventions) only assessed FV intake immediately post-intervention [50, 54–56]. All interventions were conducted in preschool settings; three studies (five interventions) specified the use of Head Start programs for the intervention [50, 55, 56]. Only four of nine studies reported the mean age of the participants, which ranged from 4.1 to 4.9 years. The remaining studies reported the proportion of 2–3 and 4–5 year olds [50, 56] or were not permitted to record the children’s ages [53].

Intervention components used across studies were nutrition education [53–56], changing the feeding environment [50, 56], peer modeling [52], and repeated exposure to FVs [53, 56]. Two interventions were classified as multi-component as they included more than one of the intervention components: Witt et al. [53] included nutrition education and repeated exposure and Smith et al. [56] included nutrition education, changing the food environment, and repeated exposure. Additionally, three interventions elicited parent involvement by sending home newsletters [56] or education materials [53] to families of participating children. No included study reported cost-effectiveness of the intervention.

Seven studies reported on directly measured fruit and/or vegetable consumption using direct observation methods of visual observation [50, 54], photo-assisted [55], or plate-waste method [52, 53] during either snack [52–54] or lunch [50, 55]. The remaining two, both by Smith and colleagues [56], estimated FV intake by measuring skin carotenoid levels using resonance Raman spectroscopy. Four interventions measured only vegetables [52, 54, 55], three interventions measured both fruits and vegetables separately [50, 53], and two interventions measured combined fruits and vegetable intake [56].

### Intervention effectiveness and study quality assessment

Overall, six out of nine interventions were able to significantly increase fruit and/or vegetable intake. Of these six, half of them were conducted in Head Start programs. Two (out of three) interventions observed an increase in fruit consumption. Four (out of seven) interventions observed an increase in vegetable consumption. One of the two interventions that measured skin carotenoids was effective at improving FV consumption. Two of the studies with no FV improvement used changing the feeding environment [50, 56] and one used peer modeling [52] as their intervention method. Unexpectedly, one intervention led to a significant decrease in FV consumption [50]. Two of these three ineffective studies were conducted in Head Start programs. The characteristics of all included studies are described in Additional file 1.

We were unable to estimate effect size for two interventions due to lack of reported standard deviations [50]. Amongst the studies with statistically significant effects, two studies [54, 55] had small effect sizes (*d* < 0.50), one [54] had a medium effect size (*d* is 0.50 to < 0.80), and the remaining two [52, 53, 56] had large effect sizes (*d* > 0.80). Amongst the studies with no statistically significant effects, both had large effect sizes (*d* > 0.80) [52, 56].

Three of the six studies (which reported on five of nine interventions) were rated as low risk of bias and the remaining four were rated as having some concerns for bias. The reasons for were no mention of pre-specified analysis plan [54] and no information regarding concealment of allocation sequence (to both researchers and subjects) prior to assignment of the intervention [53, 55]. A table reporting the risk of bias assessments is provided in Additional file 2.

### Behavior-change techniques

Intercoder reliability determined by inter-rater intraclass correlation coefficient (ICC) was “moderate” for BCTs present and absent (ICC = 0.70) and “good” for the number of BCTs used in each study (ICC = 0.77). Overall, 23 of the 93 BCTs were used in at least one intervention. Interventions used between one and ten BCTs, spanning between one and seven domains, with an average of 4.44 techniques used.

The most commonly used BCTs were “Adding objects to the environment” and “Framing/reframing”, which were both used in five of the nine interventions. The most commonly used domains (ie. used at least once within an intervention) were “Antecedents” and “Identity”, both of which were used by five interventions. The most frequently used domains (ie. total number of BCTs within this domain used within studies) were “Antecedents” and “Repetition and substitution”, whose BCTs were used nine and seven times across interventions, respectively. Two domains, “Feedback and monitoring” and “Regulation” were not used by any studies.

The BCTs and domains used in studies that did and did not effectively increase FV intake are described in Table 1. Effective studies used at least three BCTs and covered at least two domains. Two of the ineffective interventions used only the “Antecedents” domain [50, 56] and the remaining used only “Covert learning” domain [52]. No domains or BCTs were exclusive to effective or ineffective interventions.

**Table 1:** Behavior change techniques observed in included RCTs

Domain	BCT	Increased FV intake (6 interventions)	Did not increase FV intake (3 interventions)	Total (9 interventions)
Goals and planning	Action planning	1	0	1
Social support	Social support (unspecified)	1	0	1
Shaping knowledge	Instruction on how to perform a behavior	0	0	0
	Information about antecedents	1	0	1
Natural consequences	Information about health consequences	4	0	4
	Salience of consequences	3	0	3
Comparison of behavior	Demonstration of the behavior	1	0	1
Associations	Prompts/cues	1	0	1
	Exposure	1	0	1
Repetition and substitution	Behavioral practice/rehearsal	2	0	2
	Habit formation	2	0	2
	Graded tasks	1	0	1
Comparison of outcomes	Comparative imagining of future outcomes	2	0	2
Reward and threat	Material incentive (behavior)	1	0	1
	Material reward (behavior)	1	0	1
Antecedents	Restructuring the physical environment	2	1	3
	Restructuring the social environment	1	0	1
	Adding objects to the environment	3	2	5
Identity	Framing/reframing	5	0	5
Scheduled consequences	Reward approximation	1	0	1
Self-belief	Mental rehearsal of successful performance	1	0	1
Covert learning	Vicarious consequences	1	1	2

### Theoretical frameworks

Two studies [54, 56] cited separate methodology publications that were used for the Use of Theory score [57, 58]. Overall, the average Use of Theory score across interventions was 2.33 (out of 11) and ranged from 0 to 8. Four interventions [52, 53, 55, 56] mentioned a theory or model of behavior and only two of these [53, 55] linked the theory or its constructs to their intervention techniques. While the limited number of studies prohibits an empirical investigation, a narrative comparison reveals no pattern in the Use of Theory score and intervention effectiveness; the average score amongst effective and ineffective interventions was 2.17 and 2.67, respectively. In fact, the intervention with the highest Use of Theory score was ineffective [52]. The Use of Theory scoring of included interventions is described in Additional file 3.

## Discussion

We identified nine childcare- or preschool-based RCTs, reported in six different publications, that objectively measured FV intake in 2–5 year old children. Six of the nine achieved their goal of improving FV intake using the three following intervention methods: nutrition education, repeated exposure, and change in feeding environment. Two of the nine interventions had large effect sizes [55, 57], one had a moderate effect size [53], and two had small effect sizes [52, 53].

Even beyond the effect size heterogeneity, some studies reported substantial imprecision in effect size estimates. For example, in two of the studies reporting large effect sizes (*d* > 2.5), the standard errors were large and the differences did not reach the threshold for statistical significance at p < 0.05 [55, 56]. Therefore, the nonsignificant findings in these two studies may be due to small sample sizes rather than an ineffective intervention.

Five of the six effective interventions incorporated the use of nutrition education. Importantly, all nutrition education interventions were interactive for children and improved FV intake even if children were not instructed to consume FVs; merely the knowledge of the importance of FVs led to increases in consumption. These findings are in line with similar reviews that found that interventions related to “experiential learning” of nutritional concepts and healthy eating were highly effective at improving FV preference and intake in young children, compared to those relying on parental involvement or contingent reinforcement [59], and especially if they contain multiple components or strategies [60, 61].

Repeated exposure was used alongside nutrition education in two interventions, both of which were effective at improving FV intake. The use of repeated exposure to achieve behavior change stems from the Theory of Mere Exposure [62], which predicts that repeatedly exposing children to certain things, including eating healthy foods, will make them more likely to engage in that behavior in the future [63, 64]. What this theory fails to account for is that dietary behaviors are not dictated solely by liking of foods. Rather, they are driven by the complex interaction of food preferences, appetite, and external influences including, but not limited to, peer influence and pressure from a caregiver [65]. Unfortunately, as with nutrition education, no intervention in this review solely used repeated exposure, so it cannot be determined whether this intervention method is able to increase FV intake when utilized without additional components.

Based on our narrative synthesis, changing the feeding environment was not consistently effective. The only manipulation of the feeding environment that was effective at improving fruit, but not vegetable, intake was serving FV five minutes before the rest of the meal [50]. Conversely, neither providing pre-portioned meals [50], nor providing FVs for children to take home were able to improve FV intake [56]. This suggests that the low FV intake amongst preschoolers is likely not improved solely by making them more available; rather, as suggested by these findings, an additional component, such as nutrition education, may be required to improve intake [56]. In summary, these findings support the notion that many different forms of FV interventions may be effective at improving intake in preschoolers, although additional research is needed to confirm the findings.

The observation that studies with no significant improvement in FV intake used fewer BCTs is similar to the findings of other reviews evaluating BCTs and obesity-related behaviors in children [42, 66]. The BCTs used in the studies that effectively improved FV intake are similar with those reported in the review by Hendrie et al. [42]. We also observed the use of the “Framing/reframing” BCT within effective studies in this review, possibly due to different age groups (preschoolers versus all children) or the use of different versions of the BCT taxonomy. Finally, while “Restructuring the physical environment” and “Adding objects to the environment” were used in studies that both did and did not observe improvements in FV intake, the studies that exclusively used one or both of these BCTs observed no increase in FV intake, suggesting that using these two BCTs alone or in tandem is insufficient and that solely manipulating the food environment should be used with caution. Overall, our findings suggest both that no single BCT or domain must be included and no single BCT identified will certainly lead to observed improvements in FV intake in preschoolers.

The lack of association between the use of theory within interventions and their effectiveness is in line with the findings of some reviews [67–69], but opposed the findings of other reviews [70, 71]. The differences observed in the Use of Theory scores between studies likely lie in the expertise of the researchers publishing the data; the researchers with psychological backgrounds will likely be more inclined to base interventions on theory, whereas those primarily in the dietetics or physiology field may not. Regardless, most intervention studies inherently target constructs of behavioral theories, even if they are not explicitly linked, evidenced by the use of similar methods. Hence, although interventions may have been informed by theory, the authors may not have explicitly reported the theory used, and therefore received a lower Use of Theory score. Amongst the included studies, Social Cognitive Theory was the most commonly mentioned theory, in line with a similar review of obesity-preventing interventions in children [72]. Nonetheless, although rarely practiced, designing a behavior change intervention that is comprehensively informed by behavior change theory is agreed on to be a valuable factor in designing interventions aimed at changing behavior [38, 67, 68, 70].

This review differs from similar reviews in the number of included studies. This is primarily due to its narrow inclusion criteria for studies; we only included RCTs that objectively measured FV intake and were conducted in the US. This distinction was intentional to highlight the need for objectively measured outcomes in evaluating dietary interventions [18, 19]. Importantly, the findings of this review pertain only to studies in the US and may not be generalizable to other countries. The small number of studies fulfilling the inclusion criteria, and the heterogeneity in measured outcomes, precluded this review from meta-analysis. Heterogeneity in effect sizes was likely partially due to the difference in the selected outcome measures; the two largest effect sizes were observed in studies that measured skin carotenoid levels [55], and the third study measured grams of vegetable intake of a snack of bell peppers and cheerios [56]. The remainder of the studies measured pieces of FV [53] or grams [54] of FVs consumed during lunch or percent of FVs consumed (of total amount served) during lunch [57]. Hence, comparison of the effect sizes in these studies should be conducted with caution. Another consequence of a limited number of studies is the lack of inferential testing of the role of theory and BCT. As observed in larger reviews that were able to empirically test the role of theory and BCTs, this information is valuable to understanding children’s eating behavior in the US. Additionally, the evaluation of theory and BCT use is limited by the use of only published materials; authors were not contacted for additional information regarding their use of theoretical frameworks and the relevant constructs. Finally, while this review aimed to examine the cost-effectiveness of included studies, no studies provided this information. These limitations should be considered within the strengths of this systematic review, including the inclusion of only study designs that objectively measure the effects of interventions within a very specific setting and population. Importantly, this review highlights the need for further research in this field to allow for a more pragmatic evaluation in future reviews.

### Implications for future research

In addition to the need for future studies to address the limitations of this systematic review, the reviewed FV interventions and studies also highlight several opportunities for future research. Firstly, although it was not one of our primary outcomes, we found that only one study included an acclimation period to familiarize children with the research staff, methods, and changes to the eating environment. The lack of run-in time in the remaining studies may have increased the risk of the Hawthorne and placebo effects, which suggest that behavior may be altered due to the knowledge of being observed [73], particularly in studies that were implemented by unfamiliar researchers. To overcome this limitation, future studies should include an acclimation period to introduce researchers to the children and establish familiarity with equipment and methods, unless FV intake data is collected covertly. This suggestion may also mitigate any changes in eating behavior caused by the novelty of certain FVs or just novelty of being part of an intervention. Similar to studies in adults, children may be more inclined to consume FVs immediately following a nutrition education intervention on the benefits of FVs or have experienced associative conditioning from characters or books [74]. There is also need for studies with longer-term outcome measurements (ie. follow-up) to evaluate whether an intervention effect is sustainable beyond the time of direct exposure [53, 75], a critical component of all public health improvement [76]. Only two of the identified studies in this review included a follow-up period (one week and three months) [52, 53], thus, it is unknown whether the reported short-term changes in eating behavior continued [76]. This is an important aspect because only behavior changes that are maintained over time and after the children have returned to their usual environment are meaningful contributors to the effort to improve children’s diet quality. Future studies should also consider different levels of FV influence across the socioecological model (e.g. peers, family members) and potential moderators of FV intake (e.g. race, SES) to provide a more comprehensive understanding of FV intake behaviors [40, 77–79]. It is also important to consider study feasibility, acceptability, and risk of unintended impacts on children, staff, or families [80, 81], as only four of the included studies reported these effects [55, 82–84]. Evaluation of these effects in future studies may elucidate the factors associated with these undesirable or unintended effects to mitigate their impact.

Future research may also benefit from using a factorial study design that compares both different intervention components and different BCTs. These BCTs should also be placed in various contexts, such as within different intervention components, to ascertain whether the BCT or the intervention component is at play, and in heterogenous populations to explore whether different populations, or different characteristics, may respond differently to BCTs or intervention components.

## Conclusion

In conclusion, this systematic review highlights the existing evidence on RCT interventions implemented in preschools and childcare centers to improve FV intake in preschool-age children in the United States. Although only nine interventions were identified, the most consistent evidence observed is that inclusion of nutrition education components were consistently effective at improving FV intake. Studies that manipulated the feeding environment, by providing pre-portioned meals at preschool or sent FVs for children to take home, but did not directly educate children, produced inconsistent results including decreases in FV intake. Further, there was no observable pattern between the use of theoretical frameworks or BCTs and effectiveness of the studies. While several studies have shown promising results, this review highlights key gaps in this field: there is a need for more studies to test FV interventions in US childcare settings that 1) use robust designs, such as RCTs that use objective measures of dietary intake, 2) directly compare intervention components and BCTs using a factorial model, 3) explicitly report their use of theoretical frameworks, and 4) include follow-up measures to assess long-term behavior change, to determine the most effective methods to reduce the deficiency in FV intake amongst young children in the United States.

## Supplementary Information

**Additional file 1.** 

**Additional file 2.** 

**Additional file 3.** 

## Data Availability

Not applicable.

## Abbreviations

FV: Fruit and vegetable
US: United States
BCT: Behavior change technique
PRISMA: Preferred Reporting Items for Systematic Reviews and Meta-Analyses
RCT: Randomized controlled trial
ICC: Intraclass correlation coefficient
SCT: Social Cognitive Theory
CACFP: Child and Adult Care Food Program
