# Supplementary material for: Preschool- and childcare center-based interventions to increase fruit and vegetable intake in preschool children in the United States: a systematic review of effectiveness and behavior change techniques
Source: Int J Behav Nutr Phys Act. 2023 Jun 3;20:66. doi: 10.1186/s12966-023-01472-8 (PMC10239084; doi:10.1186/s12966-023-01472-8)
Supplement: Supplementary file 1 — Additional file 1. [file 12966_2023_1472_MOESM1_ESM.docx]

Table 1. Summary of Randomized Controlled trials Included in this Systematic Review.

| Study; design; intervention name and classification | Risk of Bias | Setting | Duration of intervention | Total sessions | Follow-up | Participants | F, V, or FV | Delivery of intervention | Intervention | Comparator | Use of theory score | Outcomes measured | Results |
| --- | --- | --- | --- | --- | --- | --- | --- | --- | --- | --- | --- | --- | --- |
| Gripshover, 2013a; RCT; New Theory for Nutrition; nutrition education | Some concerns | Preschool | 10-12 weeks | 22 | None | T: n=30,  Mean age=4.8 years,  51% girls | V | Researchers | Conceptual framework "new theory for nutrition" for understanding nutrition using 5 storybooks. | No treatment | 0 | Pieces of vegetables consumed during snack time, measured using visual observation | Children significantly increased their vegetable intake after the intervention (5.27 pieces) compared to no change in the control group.  Mean difference in change from baseline between groups is 5.37 pieces; t(38)=2.28, p=0.03  Cohen’s d=0.74 |
|  |  |  |  |  |  | C: n=29,  Mean age=4.6 years,  51% girls |  |  |  |  |  |  |  |
| Gripshover, 2013b; RCT; New Theory for Nutrition; nutrition education | Some concerns | Preschool | 10-12 weeks | 22 | None | T: n=53,  Mean age=4.9 years,  52% girls | V | Researchers | Conceptual framework "new theory for nutrition" for understanding nutrition using 5 storybooks. | USDA’s Team Nutrition materials | 0 | Pieces of vegetables consumed during snack time, measured using visual observation | Children significantly increased their vegetable intake after the intervention (6.15 pieces) compared to no change in the control group.  Mean difference in change from baseline between groups is 4.07 pieces; t(88)=2.06, p=0.04  Cohen’s d=0.44 |
|  |  |  |  |  |  | C: n=50,  Mean age=4.7 years,  52% girls |  |  |  |  |  |  |  |
| Harnack, 2012a; cross-over RCT; feeding environment | Low | Head Start | 2 weeks | 10 | None | N=53,  27 2-3 year olds; 26 4-5 year olds | FV | Classroom teachers | FVs were served 5 minutes prior to the rest of the meal, with the remaining portions of the meal served traditional family-style. | Typical family-style meals | 0 | Grams of FVs consumed during lunch, measured using visual observation and common household measuring tools. | Fruit: Children significantly increased their fruit intake after the intervention (0.08 cup-eq) compared to no change in the control condition; mean difference in change between groups: p<0.01  Vegetable: No difference in change in vegetable intake between groups; p>0.05 |
| Harnack, 2012b; cross-over RCT; feeding environment | Low | Head Start | 2 weeks | 10 | None |  | FV | Classroom teachers | ­­Children were served provider-portioned meals consistent with the CACFP guidelines | Typical family-style meals | 0 | Grams of FVs consumed during lunch, measured using visual observation and common household measuring tools. | Fruit: Children significantly decreased their fruit intake after the intervention (-0.07 cup-eq) compared to no change in the control condition; mean difference in change between groups: p<0.001  Vegetable: Children significantly decreased their vegetable intake after the intervention (-0.03 cup-eq) compared to no change in the control condition; mean difference in change between groups: p<0.01 |
| Nicklas, 2017; RCT; nutrition education | Some concerns | Head Start | 4 weeks | 20 | None | T: n=128, mean age 4.4 years, 51% girls | V | Classroom teachers and parents | Before lunch each day, children watched 1 of 4 DVDs theater-based puppet shows that incorporated encouragement, rationale, reinforcement, and role modeling. Children were also sent home with a copy of the DVDs, pamphlet (with positive feeding practices), and ingredients to prepare a vegetable snack. | No treatment | 7 | Vegetable consumption during normal lunch meals, measured using digital photography-assisted direct observation and plate-waste method. | Children significantly increased their vegetable intake after the intervention (13.8g) compared to no change in the control group.  Mean difference in change from baseline between groups is 11.9g; p=0.022  Cohen’s d=0.28 |
|  |  |  |  |  |  | C: n=125, mean age 4.4 years, 51% girls |  |  |  |  |  |  |  |
| Smith, 2020a; RCT; Harvest for Healthy Kids; feeding environment | Low | Head Start | 8 weeks | 40 | None | T: n=61, 56% girls | FV | Parents | Children received high-carotenoid FVs to take home | No treatment | 0 | ­­Skin carotenoid levels measured using Resonance Ramen Spectroscopy | No difference in change in skin carotenoids from baseline between the intervention (4887) and control (2623) groups.  Mean difference in change from baseline between groups is 2264, p=0.10  Cohen’s d=2.89 |
|  |  |  |  |  |  | C: n=66, 55% girls |  |  |  |  |  |  |  |
| Smith, 2020b; RCT; Harvest for Healthy Kids; nutrition education, change in feeding environment, and repeated exposure | Low | Head Start | 8 weeks | 40 | None | T: n=82, 45% girls | FV | SNAP-Ed staff members | 30-min daily SNAP-Ed classroom curriculum (Harvest for Healthy Kids) which included a story and picture cards (with FV characters), hands-on food preparation activity, and taste-testing, *plus* children received high carotenoid FVs and parent nutrition education materials to take home. | No treatment | 4 | ­­Skin carotenoid levels measured using Resonance Ramen Spectroscopy | Skin carotenoid levels increased significantly more in the intervention (7834) versus the control (2623) group.  Mean difference in change from baseline between groups is 5211; F(2,206)=12.961, p<0.001  Cohen’s d=7.14 |
|  |  |  |  |  |  | C: n=66, 55% girls |  |  |  |  |  |  |  |
| Staiano, 2020; RCT; Copy-Kids Eat Fruits and Vegetables; peer modeling | Low | Preschool | 1 day | 1 | 7 days | T: n=14, mean age 4.5 years, 50% girls | V | Researchers | On day 1, children were presented with a snack while watching a 7.5min "Copy-Kids" DVD clip designed to encourage positive eating habits, where similarly-aged toddlers happily ate and vocally interacted with the foods (bell peppers). The snack procedure was repeated on days 2 and 7, without any video. | “Brush Teeth” DVD segment | 8 | Consumption of bell peppers during snack time, measured using plate-waste method | No difference in vegetable intake following the intervention video (11.01g), compared to the control video (-3.75g); p>0.05  Cohen’s d=3.37 |
|  |  |  |  |  |  | C: n=14, mean age 4.1 years, 50% girls |  |  |  |  |  |  |  |
| Witt, 2012a; RCT; Color Me Healthy; nutrition education and repeated exposure | Some concerns | Childcare center | 6 weeks | 18 | None | T: n=165, majority 4-5 years old | FV | Classroom teachers | 2 circle-time lessons and 1 imaginary trip (15-30min each) consisting of interactive learning opportunities on physical activity and healthy eating. Toolkit includes picture cards, posters, music CD, mostly focusing on FVs of different colors, and interactive taste-tests. Interactive take-home activities were also provided. | No treatment | 2 | Percentage of FV consumed during snack time (Fs and Vs served on separate days and Vs served with fat-free ranch), measured using plate-waste method | Fruits: Children consumed a greater percentage of fruits following the intervention (31.20%) compared to the control group (-8%). Mean difference in change between groups is 39.2%; F(1,149)=62.26, p<0.001  Cohen’s d=1.29  Vegetables: Children consumed a greater percentage of vegetables following the intervention (24.20%) compared to the control group (-2.4%). Mean difference in change between groups is 26.6%; F(1,120)=24.14, p<0.001  Cohen’s d=0.90 |
|  |  |  |  |  |  | C: n=98, majority 4-5 years old |  |  |  |  |  |  |  |
| Witt, 2012b; RCT; Color Me Healthy; nutrition education and repeated exposure | Some concerns | Childcare center | 6 weeks | 18 | 3 months | T: n=165, majority 4-5 years old | FV | Classroom teachers | 2 circle-time lessons and 1 imaginary trip (15-30min each) consisting of interactive learning opportunities on physical activity and healthy eating. Toolkit includes picture cards, posters, music CD, mostly focusing on FVs of different colors, and interactive taste-tests. Interactive take-home activities were also provided. | No treatment | 2 | Percentage of FV consumed during snack time (Fs and Vs served on separate days and Vs served with fat-free ranch), measured using plate-waste method | Fruits: Children consumed a greater percentage of fruits following the intervention (20.80%) compared to the control group (-1.4%). Mean difference in change between groups is 22.2%; F(1,149)=17.41, p<0.001  Cohen’s d=0.68  Vegetables: Children consumed a greater percentage of vegetables following the intervention (33.10%) compared to the control group (-1.6%). Mean difference in change between groups is 34.5%; F(1,120)=43.41, p<0.001  Cohen’s d=1.20 |
|  |  |  |  |  |  | C: n=98, majority 4-5 years old |  |  |  |  |  |  |  |
