# Supplementary material for: Preschool- and childcare center-based interventions to increase fruit and vegetable intake in preschool children in the United States: a systematic review of effectiveness and behavior change techniques
Source: Int J Behav Nutr Phys Act. 2023 Jun 3;20:66. doi: 10.1186/s12966-023-01472-8 (PMC10239084; doi:10.1186/s12966-023-01472-8)
Supplement: Supplementary file 2 — Additional file 2. [file 12966_2023_1472_MOESM2_ESM.docx]

Additional file 2. Risk of Bias Assessment of Studies Included in this Systematic Review

| Study; design; intervention name and classification | Domain 1a. Randomization process | Domain 1b: Risk of bias arising from the timing of identification or recruitment of participants | Domain 2. Deviations from intended interventions | Domain 3. Missing outcome data | Domain 4. Measurement of the outcome | Domain 5. Selection of the reported result | Domain 6. Overall Bias |
| --- | --- | --- | --- | --- | --- | --- | --- |
| Gripshover, 2013; RCT; New Theory for Nutrition | Low | Low | Low | Low | Low | Some concerns | Some concerns |
| Harnack, 2012; cross-over RCT | Low | Low | Low | Low | Low | Low | Low |
| Nicklas, 2017; RCT | Some concerns | Low | Low | Low | Low | Low | Some concerns |
| Smith, 2020; RCT; Harvest for Healthy Kids | Low | Low | Low | Low | Low | Low | Low |
| Staiano, 2020; RCT; Copy-Kids Eat Fruits and Vegetables | Low | Low | Low | Low | Low | Low | Low |
| Witt, 2012; RCT; Color Me Healthy | Some concerns | Low | Low | Low | Low | Low | Some concerns |
