# Supplementary material for: Preschool- and childcare center-based interventions to increase fruit and vegetable intake in preschool children in the United States: a systematic review of effectiveness and behavior change techniques
Source: Int J Behav Nutr Phys Act. 2023 Jun 3;20:66. doi: 10.1186/s12966-023-01472-8 (PMC10239084; doi:10.1186/s12966-023-01472-8)
Supplement: Supplementary file 3 — Additional file 3. [file 12966_2023_1472_MOESM3_ESM.docx]

Additional file 3. Use of Theory Scoring of Studies Included in this Systematic Review

| Study; design; intervention name and classification | Use of Theory coding scheme | | | | | | | | | | | Overall Use of Theory Score |
| --- | --- | --- | --- | --- | --- | --- | --- | --- | --- | --- | --- | --- |
|  | Theory/ model of behavior mentioned | Targeted construct mentioned as predictor of behavior | Intervention based on single theory | Theory/ predictors used to select recipients for the intervention | Theory/ predictors used to select/develop intervention techniques | Theory/ predictors used to tailor intervention techniques to recipients | ALL intervention techniques are explicitly linked to at least one theory-relevant construct/predictor | At least one of the intervention technique is explicitly linked to at least one theory-relevant construct/predictor | Group of techniques are linked to a group of constructs/ predictors | All theory-relevant constructs/ predictors are explicitly linked to at least one intervention technique | At least one theory-relevant constructs/ predictors is explicitly linked to at least one intervention technique |  |
| Gripshover, 2013a; RCT; New Theory for Nutrition; nutrition education |  |  |  |  |  |  |  |  |  |  |  | 0 |
| Gripshover, 2013b; RCT; New Theory for Nutrition; nutrition education |  |  |  |  |  |  |  |  |  |  |  | 0 |
| Harnack, 2012a; cross-over RCT; feeding environment |  |  |  |  |  |  |  |  |  |  |  | 0 |
| Harnack, 2012b; cross-over RCT; feeding environment |  |  |  |  |  |  |  |  |  |  |  | 0 |
| Nicklas, 2017; RCT; nutrition education | x | x | x |  | x | x |  | x | x |  |  | 7 |
| Smith, 2020a; RCT; Harvest for Healthy Kids; feeding environment |  |  |  |  |  |  |  |  |  |  |  | 0 |
| Smith, 2020b; RCT; Harvest for Healthy Kids; nutrition education, change in feeding environment, and repeated exposure | x | x | x |  | x |  |  |  |  |  |  | 4 |
| Staiano, 2020; RCT; Copy-Kids Eat Fruits and Vegetables; peer modeling | x | x | x |  | x |  | x | x | x |  | x | 8 |
| Witt, 2012; RCT; Color Me Healthy; nutrition education and repeated exposure | x | x |  |  |  |  |  |  |  |  |  | 2 |
